# TGF-β signaling in lymphatic vascular vessel formation and maintenance

**DOI:** 10.3389/fphys.2022.1081376

**Published:** 2022-12-15

**Authors:** Fumiko Itoh, Tetsuro Watabe

**Affiliations:** ^1^ Laboratory of Stem Cells Regulations, Tokyo University of Pharmacy and Life Sciences, Tokyo, Japan; ^2^ Department of Biochemistry, Graduate School of Medical and Dental Sciences, Tokyo Medical and Dental University (TMDU), Tokyo, Japan

**Keywords:** TGF-β, lymphatic vessel, endothelial cell, Prox1, tumor metastasis

## Abstract

Transforming growth factor (TGF)-β and its family members, including bone morphogenetic proteins (BMPs), nodal proteins, and activins, are implicated in the development and maintenance of various organs. Here, we review its role in the lymphatic vascular system (the secondary vascular system in vertebrates), which plays a crucial role in various physiological and pathological processes, participating in the maintenance of the normal tissue fluid balance, immune cell trafficking, and fatty acid absorption in the gut. The lymphatic system is associated with pathogenesis in multiple diseases, including lymphedema, inflammatory diseases, and tumor metastasis. Lymphatic vessels are composed of lymphatic endothelial cells, which differentiate from blood vascular endothelial cells (BECs). Although TGF-β family signaling is essential for maintaining blood vessel function, little is known about the role of TGF-β in lymphatic homeostasis. Recently, we reported that endothelial-specific depletion of TGF-β signaling affects lymphatic function. These reports suggest that TGF-β signaling in lymphatic endothelial cells maintains the structure of lymphatic vessels and lymphatic homeostasis, and promotes tumor lymphatic metastasis. Suppression of TGF-β signaling in lymphatic endothelial cells may therefore be effective in inhibiting cancer metastasis. We highlight recent advances in understanding the roles of TGF-β signaling in the formation and maintenance of the lymphatic system.

## Introduction

**FIGURE 1:**
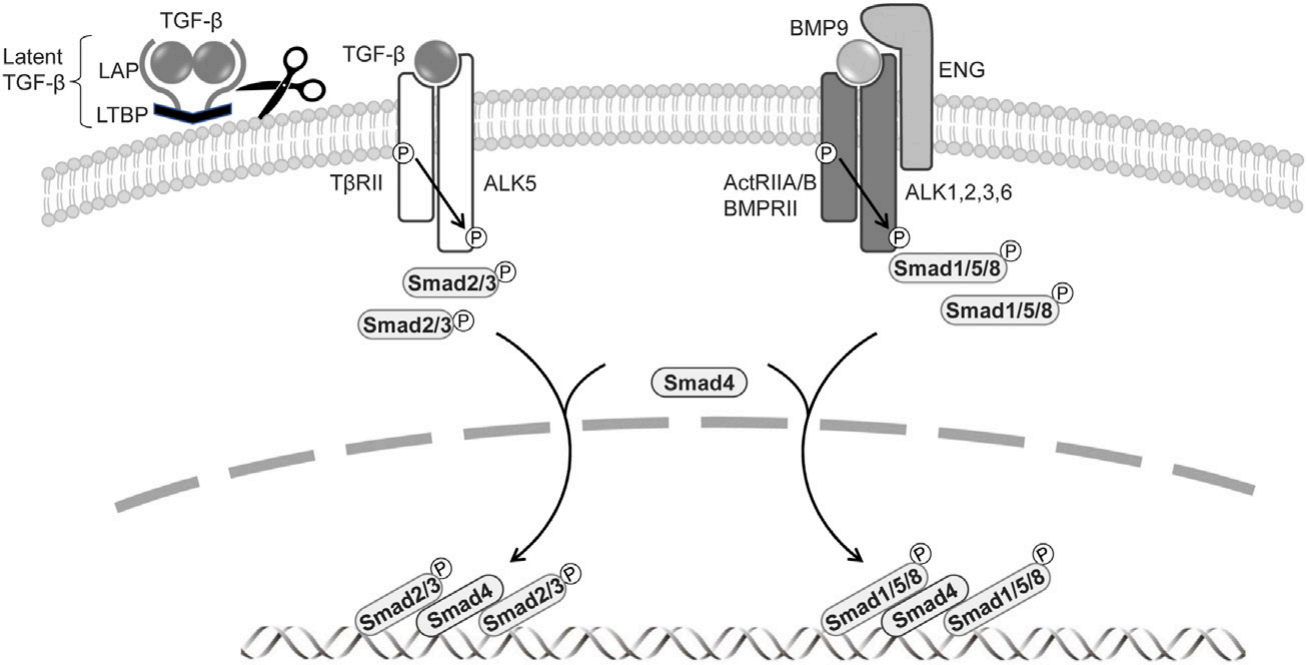
Signal transduction by TGF-β family members in BECs and LECs.The signaling is mediated through a heteromeric complex of specific type I and type II serine/threonine kinase receptors. TGF-β is produced as a precursor by a single gene and then proteolytically cleaved into three fragments: latent TGF-β-binding protein (LTBP), an N-terminal latency-associated protein (LAP), and an active C-terminal cytokine region. Activated TGF-β interacts with TβRII and ALK5. BMPs signal *via* ActRIIA/B, BMPRII, and type I receptors ALK1, 2, 3, and 6. The co-receptor endoglin and ALK1, exclusively expressed on endothelial cells, are involved in BMP9 signaling. TGF-βs phosphorylate of Smad2 and Smad3, and BMPs induce phosphorylation of Smad1, 5, and 8. Activated Smads form heteromeric complexes with Smad4, which translocate to the nucleus, where they regulate the target gene expressions.

The TGF-β superfamily, containing more than 30 cytokines in mammals, comprises secreted dimeric proteins that induce pleiotropic effects by regulating cell proliferation, differentiation, and survival (Morikawa, et al., 2016). TGF-β signaling operates *via* cell membrane receptors and intracellular effector proteins. TGF-β1, its best-characterized member, is initially produced as a precursor by a single gene. This is then proteolytically cleaved *via* the proprotein-converting enzyme furin into three fragments: latent TGF-β-binding protein (LTBP), an N-terminal latency-associated protein (LAP), and an active C-terminal cytokine region (Figure 1). The dimerized mature TGF-β and LAP domains are maintained by non-covalent bonds, interact covalently with LTBP, and are retained in the extracellular matrix (ten Dijke and Arthur 2007). Retention of the latent TGF-β in the extracellular matrix is important for effective TGF-β bioavailability and signal transduction (Robertson, et al., 2015; Robertson and Rifkin 2016). Although proteases activate latent TGF-β (Abe, et al., 1998), it is also activated by non-protease factors, including thrombospondin (TSP-1) (Ribeiro, et al., 1999), neuropilin-1 (Nrp1) (Glinka and Prud’homme 2008), and by environmental factors such as acidity, heat, shear stress, and ultraviolet radiation (Robertson, et al., 2015). The TGF-β family includes bone morphogenetic proteins (BMPs); the role of BMPs in the vascular system has been discussed elsewhere (Goumans, et al., 2018).

TGF-β family signaling is initiated by the binding of a ligand to a complex of two serine/threonine kinase receptors, five type-II receptors, and seven type-I receptors (also known as activin receptor-like kinases, ALKs) (Itoh, et al., 2014; Heldin and Moustakas 2016). In canonical signaling, TGF-β signals *via* the TGF-β type-II receptor (TβRII) and ALK5, while BMPs transduce signals *via* the BMP type-II receptor (BMPRII) or activin type-II receptor (ActRIIA/B) and ALK1, ALK2, ALK3, and ALK6. The activated type-I receptor phosphorylates receptor-regulated Smads (R-Smads) at two C-terminal serine residues. TGF-β induces the phosphorylation of Smad2 and Smad3, whereas BMP mediates the activation of Smad1, Smad5, and Smad8/Smad9 (Katagiri and Watabe 2016). The two phosphorylated R-Smads form ternary complexes with the common partner Smad (Co-Smad) Smad4, which then enters the nucleus, where it acts as a transcriptional factor to regulate TGF-β target-gene expression (Figure 1). TGF-β also transduces signals *via* a number of non-canonical pathways, inducting mitogen-activated protein kinases (MAPKs), phosphoinositide-3-kinase (PI3K), and Rho like GTPases (Zhang 2017). The TGF-β pathway includes co-receptors, namely the TβRIII receptors betaglycan and endoglin (Bilandzic and Stenver 2011; ten Dijke, et al., 2008); while these have large extracellular and small intracellular domains that lack enzymatic activity, they regulate TGF-β access to its specific receptors. Betaglycan is required for TGF-β2 to bind to TβRII (Rotzer, et al., 2001), and endoglin and betaglycan enhance BMP9 and BMP10 signaling (David, et al., 2007).

Unlike factors such as vascular endothelial growth factor (VEGF), which have prominent effects on endothelial cells, TGF-β has cell type-specific and context-dependent effects on many cell types *via* ubiquitously expressed receptors (David and Massague 2018). Misregulation of TGF-β signaling in humans leads to vascular pathology and cardiovascular diseases such as vascular remodeling of the retina (retinopathy), arteriovenous malformations (AVM), aneurysms, atherosclerosis, and valvular heart disease (Akhurst and Hata 2012). Studies of various knockout mice have shown that the TGF-β signaling pathways are important for maintaining blood vascular function (Goumans and Mummery 2000). Although lymphatic system research is not as advanced as blood vessel research, substantial progress has been made by identifying specific markers for *in vitro* and *in vivo* analysis of lymphatic endothelial cells (LECs). Accordingly, our understanding of the role of TGF-β signaling in LECs is increasing. In this review, we highlight recent insights into the function of TGF-β in lymphatic vessels, and discuss the contribution of TGF-β signaling dysregulation to tumor progression.

## Lymphatic system

The circulatory system comprises the vascular system, in which the blood circulates, and the lymphatic system, comprising lymphatic capillaries and collecting lymphatic vessels. In these systems, the lumen is covered by BECs or LECs, respectively (Karpanen and Alitalo 2008). Several signaling pathways involved in angiogenesis play important roles in lymphangiogenesis. Among them, many growth factors have similar effects on BECs and LECs. A potent growth factor foe endothelial cells is VEGF family, with VEGF-A/VEGF receptor 2 (VEGFR2) signaling plays a central role in BECs and VEGF-C/VEGFR3 singling in LECs. Angiopoietin-2 (Angpt2), however, has opposite effects on its receptor Tie2 signaling in BECs and LECs (Souma, et al., 2018). Unlike the circulatory system, the lymphatic system undergoes afferent flow, whereby lymphatic fluid drains from small capillaries into the pre-collecting and collecting vessels, flows through the lymph nodes, and finally joins the vascular circulation at the junction with the subclavian vein. The primary function of lymphatic vessels is to transport body fluids and cellular components (Brakenhielm and Alitalo 2019). Lymphatic vessels maintain homeostasis by balancing tissue fluids throughout the body, coordinating immune surveillance, and maintaining lymphangiogenesis in response to environmental conditions.

In mammals, lymphatic vessels arise from progenitor cells that bud off from the lateral surfaces of cardinal vein endothelial cells during embryonic development (Escobedo and Oliver 2016) (Oliver, et al., 2020). Recent studies have also demonstrated that LECs also develop from *de novo* production from non-venous sources such as mesenchymal cells, hemogenic endothelium, musculoendotuelial progenitor cells, and craniopharyngeal mesoderm (Stone and Stainier 2019) (Maruyama, et al., 2022). The expression of Sox8 and COUP-TFII/Nr2f2 in venous endothelial cells initiates the expression of Prospero homeobox transcription factor-1 (Prox1), which is essential for the development and maintenance of LECs (Srinivasan, et al., 2007). Prox1 expression then induces the expression of VEGFR3, which transduces VEGF-C stimuli. The progenitor cells then bud from the vein and establish the lymphatic sac. In this process, Prox1 maintains VEGFR3 gene expression, while VEGFR3 signals regulate Prox1 expression, thus generating and maintaining lymphatic endothelial progenitor cells. The lymphatic sacs give rise to LECs that depend on interactions with VEGF-C/VEGFR3; these then spread to the surrounding area, and gradually expand into a network of lymphatic vessels. Lympatic vessel endothelial hyaluronan receptor 1 (LYVE-1), a type I transmembrane glycoprotein, is expressed both by lymphatic endothelial progenitor cells and postnatal macrophages, which are known to stimulate lymphangiogenesis (Cho, et al., 2007; Ran and Montgomery 2012). Although macrophages may differentiate into LECs, they primarily stimulate the proliferation of existing LECs by secreting growth factors such as VEGF-C (Adams and Alitalo 2007).

## TGF-β signaling in endothelial cells

ALK1 (a BMP9 and BMP10 receptor) and endoglin participate in endothelial cell-specific TGF-β family signaling. While both receptors participate in the phosphorylation of Smad1, Smad5, and Smad8, ALK1 can also bind TGF-β with low affinity, and thus induce Smad2 and Smad3 activation. Mutations in these genes involved in TGF-β family signaling are causally related to hereditary hemorrhagic telangiectasia, and mutations in BMPR2 and Smad4 cause pulmonary arterial pulmonary hypertension, indicating that the TGF-β/BMP signaling plays an important role in maintaining vascular function.

### How TGF-β affects vessel formation

TGF-β and BMP transduce their signals *via* different R-Smads, playing complex roles. In cultured LECs, TGF-β treatment suppresses Prox-1 and LYVE1 expression (Yoshimatsu, et al., 2020). In mouse embryonic stem cell-derived VEGFR2^+^-endothelial cells, TGF-β inhibits the lymphatic endothelium and downregulates related markers, including COUP-TFII and Sox18. Similarly, BMP9 inhibits Prox1 expression *in vitro*. The BMP9/ALK1 pathway induces differentiation from an LEC phenotype to a BEC phenotype (Yoshimatsu, et al., 2013). Contradictory, it has been reported that stimulation with TGF-β2 inhibits proliferation and increases the expression of VEGFR3 and Nrp2, which participate in lymphatic budding (James, et al., 2013). Hence, *in vitro*, the effects of TGF-β family members on endothelial cells can be depend on several factors, including the original ligand concentration, serum composition, cell density, and the types of TGF-β receptors expressed on the membrane (Goumans and ten Dijke 2018).

**FIGURE 2:**
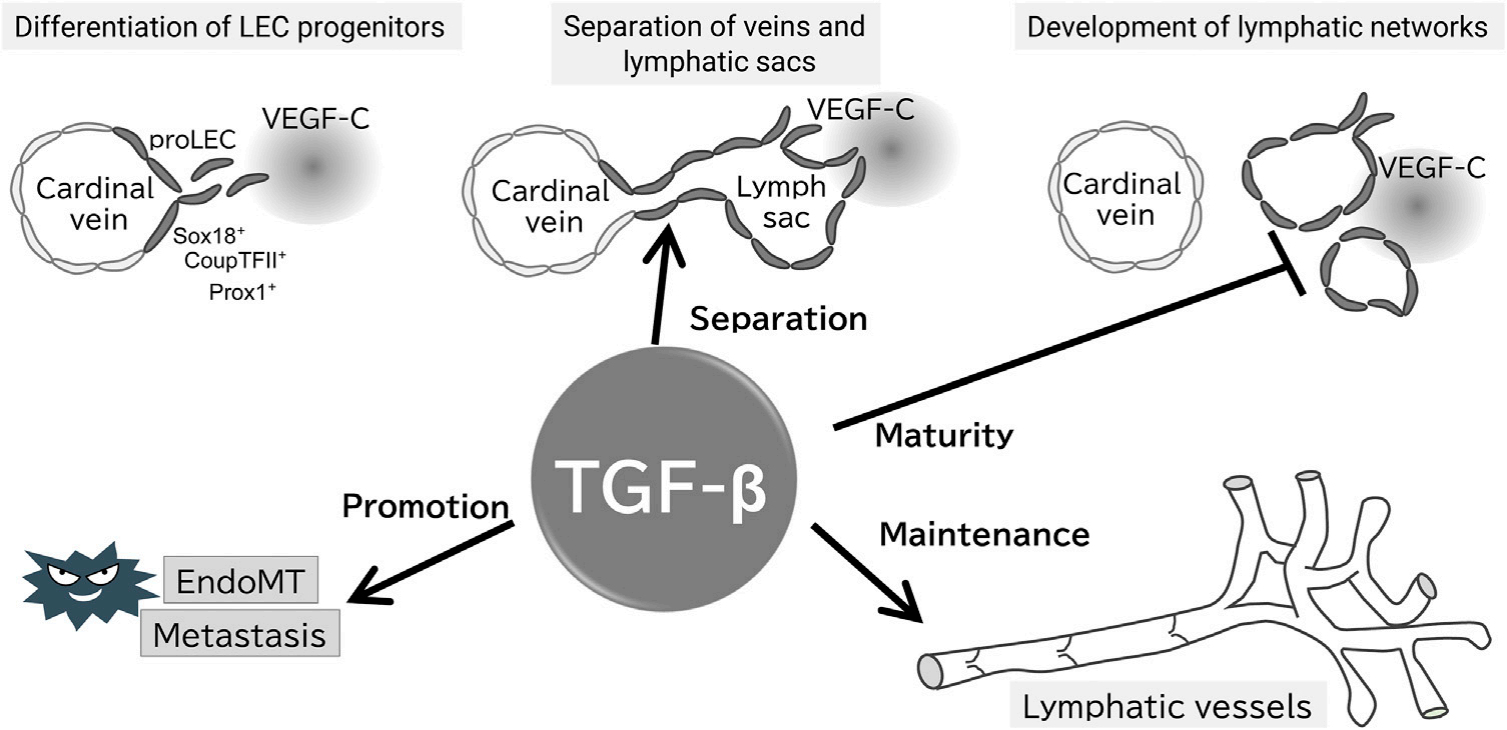
Proposed working model of TGF-β signaling in the formation and maintenance of the lymphatic system. Evidence for the presence of each step of the proposed model is provided in the text; TGF-β does not affect the early development of lymphatic vessels, whereas is important for their separation from veins, maturation, and maintenance of lymphatic function. TGF-β also promotes tumor lymphatic metastasis *via* EndoMT of LECs.

In mice, targeted deletion of ALK1, ALK5, TβRII, or endoglin results in embryonic lethality due to angiogenesis failure, leading to similar phenotypes (Goumans and Mummery 2000). Tie2, a receptor for angiopoietins, is expressed in BECs and hematopoietic cells. Embryos lacking TGF-β signaling in a Tie2 promoter-dependent manner (Smad2^fl/fl^; Smad3^−/−^; Tie2-Cre) exhibit leaky vessels and die at embryonic day 12.5, even though a hierarchical vascular structure is formed. Interestingly, these mice do not exhibit lymphatic vessel abnormalities (Itoh, et al., 2012). LEC progenitors derived from the superficial venous plexus recombine highly efficiently in Tie2 promoter dependent manner, and crosses between Tie2-Cre mice and folliculin (FLCN) conditional knockout mice have been reported to show abnormalities in lymphatic vessels (Martinez-Corral, et al., 2015; Tai-Nagara, et al., 2020). Loss of FLCN promotes excessive commitment of venous endothelial cells to LECs, because FLCN prevents the accumulation and nuclear translocation of the transcription factor E3 (TFE3), which upregulates Prox1 gene expression (Tai-Nagara, et al., 2020). These results highlight that TGF-β signaling is important in the vascular maturation process, but not in LEC commitment in mice embryos, contradictory to *in vitro* analysis (Figure 2).

### How TGF-β affects vessel function

Based on *in vivo* analysis, TGF-β signaling is crucial for maintaining lymphatic vessel structure and function. Endothelium-specific loss of ALK5 or TβRII in early development reduces cutaneous lymphatic network complexity and leads to abnormal lymphatic vessel morphology (James, et al., 2013; Fukasawa, et al., 2021) (Figure 2), potentially due to the loss of TGF-β signaling-induced inhibition of growth. Treatment of chronic peritonitis with a small-molecule ALK5 inhibitor increased lymphangiogenesis. A study of TGF-β signaling in pathological lymphangiogenesis indicated the potential involvement of VEGF-C secreted by inflammatory macrophages (Oka, et al., 2008). In postnatal mice, reduced endothelial cell-specific TβRII gene expression results in abnormally dilated lymphatic vessels and reduced drainage (Fukasawa, et al., 2021). In other words, TGF-β regulates lymphatic vessel function.

TGF-β, an inducer of the epithelial-mesenchymal transition (EMT), also regulates the function of LECs *via* endothelial-mesenchymal transition (EndoMT). BECs and LECs acquire mesenchymal properties through EndoMT (Yoshimatsu and Watabe 2022). We have reported that TGF-β2 expression in dermal tissue increases during aging, inducing lymphatic vessel EndoMT through RhoA/Rock non-Smad pathway in cooperation with inflammatory signaling (Yoshimatsu, et al., 2020) (Figure 2). EndoMT, which occurs during normal cellular differentiation in cardiac development, is implicated in conditions such as cardiac fibrosis, atherosclerosis, pulmonary hypertension, and cancer (Zeisberg, et al., 2007; Hong, et al., 2018; Szulcek, et al., 2020). Therefore, targeting of EndoMT may be beneficial for the treatment of huma disorders.

### How TGF-β impacts the lymphatic vessels of the tumor

Tumors comprise cells that have diversified by accumulating genetic mutations at multiple stages, resulting in polyclonal cell populations (McGranahan and Swanton 2017). Normal epithelial cells adhere tightly to each other, maintaining the three-dimensional structure of the tissue, whereas cancer cells reduced intercellular adhesion and increased motility and invasiveness through EMT (Brabletz, et al., 2018). TGF-β, one of potent EMT inducing cytokines which include fibroblast growth factor (FGF), hepatocyte growth factor (HGF), platelet-derived growth factor (PDGF), Notch, and Wnt (Heldin, et al., 2012). Cancer cells that have left the primary tumor invade the adjacent tissue, and reach to the lumen *via* the sheet structure of vascular and lymphatic endothelial cells. The cancer cells then migrate to distant organs or locations *via* blood and lymph flow, grow into capillaries and lymph nodes in distant organs, and form new tumors (Chambers, et al., 2002). EMT-induced cancer cells show reduced expression of E-cadherin, a potent promoter of intercellular adhesion and an epithelial cell marker, and reduced expression of mesenchymal cell-specific cytoskeletal proteins such as vimentin. Other mesenchymal markers include N-cadherin, fibronectin, and smooth muscle α-actin (α-SMA). Cancer cells that have acquired mesenchymal properties exhibit increased migration and invasiveness, and enhanced stress resistance, immunosuppression, and stem cell-like properties. These properties enhance malignant transformation in cancer and participate centrally in the acquisition of chemotherapy resistance. Cancer-associated fibroblasts have been identified as a major cause of tumor growth and metastatic dissemination. TGF-β, which is abundantly produced in chronic inflammation and in the tumor microenvironment, is a potent inducer of not only for EMT but also EndoMT.

The spread of cancer cells from the primary tumor to the lymph nodes is an important determinant of patient prognosis and treatment (Vaahtomeri and Alitalo 2020). Tumor lymphangiogenesis occurs primarily around the tumor; it is unlikely to occur inside the tumor, because of the high interstitial pressure within the tumor (Padera, et al., 2004). Hence, within tumors, cancer cells are more likely to metastasize *via* blood vessels than *via* lymphatic vessels. In colorectal cancer, the abundance of lymphatic vessels peripheral to cancerous tissue is reportedly correlated with patient survival rates, because peripheral lymphatic vessel function both as drainage channels for tissue fluid and as transport routes for immune cells. The invasive front, the most advanced zone of cancer invasion, is surrounded by many functional lymphatic vessels, which serve as the starting point for lymphatic metastasis. These peritumoral lymphatic vessels promote lymphangiogenesis in response to VEGF-C produced by cancer cells (Zhang, et al., 2021), inducing lymphatic sprouting and hyperplasia around the tumor.

TGF-β signaling participates in tumor lymphangiogenesis (Oka, et al., 2008; Pak, et al., 2019) and inhibition of BMP9 by small-molecule compounds inhibits tumor lymphangiogenesis (Yoshimatsu, et al., 2013). In tumor xenografts, ALK5 inhibitors increase lymphangiogenesis (Oka, et al., 2008). Similarly, the loss of endothelial cell-specific TβRII dilates the lymphatic lumen, exacerbating tumor lymphangiogenesis (Fukasawa, et al., 2021) (Figure 2). These results indicate that TGF-β acts on tumor metastasis by regulating the structure and function of the newly created tumor lymphatic vessels. Secondary lymphedema is a common complication of cancer treatment and TGF-β1 has been shown to be increased in this disease. Inhibition of TGF-β1 has been shown to reduce the severity of lymphedema in mouse models (Baik, et al., 2022). Therefore, targeting TGF-β may lead to effective inhibition of lymphatic metastasis and lymphedema.

## Conclusion

TGF-β signaling has pleiotropic effects on endothelial cells, performing a vast array of actions under both physiological and pathological conditions. While VEGF-C/VEGFR-3 signaling is known to play a major role in lymphangiogenesis, the importance of TGF-β/BMPs is now becoming clear. Understanding the molecular mechanisms regulating LECs will lead to the development of new therapies for many LEC-associated human diseases. Nonetheless, the various context-dependent functions of TGF-β signaling remain to be clarified. In particular, future research will elucidate the complexity of TGF-β signaling, and its roles in regulating lymphatic function and cellular behavior.
